# Hospital for Sick Children, Great Ormond Street

**Published:** 1902-08-16

**Authors:** 


### HOSPITAL FOR SICK CHILDREN, GREAT ORMOND STREET.

### THE YEAR'S WORK.

There was a deficit of £6,626 on this hospital at the close of 1901, the ordinary expenditure being £18,982, and the ordinary income £12,356. Legacies amounted to £3,697, and this sum, together with the £6,000 that remained over from the magnificent total collected by "Mr. Punch" in 1900, enabled the committee to meet the deficit above mentioned, and the extraordinary expenditure, which amounted to £1,528. It is also in a great measure owing to the fresh and sympathetic interest aroused in the public mind by "Mr. Punch" that there is a further and most encouraging increase in both subscriptions and donations, that on the former amounting to £294, and on the latter to £663. King Edward's Fund gave £600; this was increased by £100 on the amount awarded the previous year, to provide, in part, for the opening and maintenance of the Goldsmiths' Ward for the treatment of whooping cough. This ward received 135 patients during the year. The increased expenditure is due to a variety of causes, among which are the addition of two resident medical officers to the staff, and of 15 nurses for the Goldsmiths' Ward; the establishment of a Bacteriological Department, and generally the advanced prices of commodities. The average cost per in-patient was 3s. 2d. less than in the previous year; that of each out-patient, 3¾d. less. The Endowment Fund amounts to a capital of £41,600, yielding an income equal to about one-sixth of the minimum endowment revenue estimated as necessary for a hospital of this size and character; and a yearly deficit of £7,000 has to be faced. The sum of £1,235 has to be raised yearly to repay the loan of £24,000 from the Prudential Assurance Company for the Nurses' House acquired by the purchase of the adjoining Hospital of St. John and St. Elizabeth. The absolute necessity for a new Out-Patients' Department was urged by Sir Henry Burdett at the annual meeting in May.

